# Nickel hexa­yttrium deca­iodide, [NiY_6_]I_10_

**DOI:** 10.1107/S1600536814009775

**Published:** 2014-05-10

**Authors:** Simon Steinberg, Gerd Meyer

**Affiliations:** a Department für Chemie, Anorganische Festkörper- und Koordinationschemie, Universität zu Köln, Greinstrasse 6, D-50939 Köln, Germany

## Abstract

Comproportionation reactions of yttrium triiodide, yttrium and nickel led to the formation of the compound [NiY_6_]I_10_, which is isostructural with the prototypical [RuY_6_]I_10_. In particular, [NiY_6_]I_10_ is composed of isolated nickel centered yttrium octa­hedra (site symmetry -1) that are further surrounded by iodide ligands to construct a three-dimensional cluster complex framework. Although this compound has been previously detected by powder X-ray diffraction techniques [Payne & Corbett (1990 ▶). *Inorg. Chem.* **29**, 2246–2251], details of the crystal structure for triclinic [NiY_6_]I_10_ were not provided.

## Related literature   

For a report of the prototypical [RuY_6_]I_10_, see: Hughbanks *et al.* (1989 ▶). For the determination of the lattice parameters of [NiY_6_]I_10_ from PXRD data, see: Payne & Corbett (1990 ▶). For a survey of isotypic structures, see: Rustige *et al.* (2012 ▶). For the synthesis of the starting material YI_3_, see: Corbett (1983 ▶); Meyer (1991 ▶). The symmetry of the refined structure was checked using the *PLATON* software package (Spek, 2009 ▶).

## Experimental   

### 

#### Crystal data  

[NiY_6_]I_10_
*M* *_r_* = 1861.17
Triclinic, 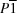

*a* = 9.4904 (7) Å
*b* = 9.4990 (7) Å
*c* = 7.5702 (5) Å
α = 97.056 (6)°
β = 105.096 (6)°
γ = 107.540 (5)°
*V* = 613.06 (8) Å^3^
*Z* = 1
Mo *K*α radiation
μ = 27.35 mm^−1^
*T* = 293 K
0.1 × 0.1 × 0.1 mm

#### Data collection  

Stoe IPDS 2T diffractometer
Absorption correction: numerical [*X-SHAPE* (Stoe & Cie, 1999 ▶) and *X-RED* (Stoe & Cie, 2001 ▶)] *T*_min_ = 0.031, *T*_max_ = 0.084
11699 measured reflections
3294 independent reflections
2873 reflections with *I* > 2σ(*I*)
*R*_int_ = 0.106

#### Refinement  

*R*[*F* ^2^ > 2σ(*F* ^2^)] = 0.034
*wR*(*F* ^2^) = 0.091
*S* = 1.05
3294 reflections
80 parameters
Δρ_max_ = 1.37 e Å^−3^
Δρ_min_ = −1.64 e Å^−3^

### 

Data collection: *X-AREA* (Stoe & Cie, 2003 ▶); cell refinement: *X-AREA*; data reduction: *X-AREA*; program(s) used to solve structure: *SHELXS97* (Sheldrick, 2008 ▶); program(s) used to refine structure: *SHELXL97* (Sheldrick, 2008 ▶); molecular graphics: *DIAMOND* (Brandenburg, 1999 ▶); software used to prepare material for publication: *SHELXL97* and local programs.

## Supplementary Material

Crystal structure: contains datablock(s) I, global. DOI: 10.1107/S1600536814009775/hp2066sup1.cif

Structure factors: contains datablock(s) I. DOI: 10.1107/S1600536814009775/hp2066Isup2.hkl

CCDC reference: 1000440

Additional supporting information:  crystallographic information; 3D view; checkCIF report

## Figures and Tables

**Table 1:** Averaged Y—Ni, Y—Y and Y—I distances (Å) in triclinic [NiY_6_]I_10_

Inter­action	Y—Ni	Y—Y	Y—I
Distance	2.649	3.746	3.143

**Fig. 1.**
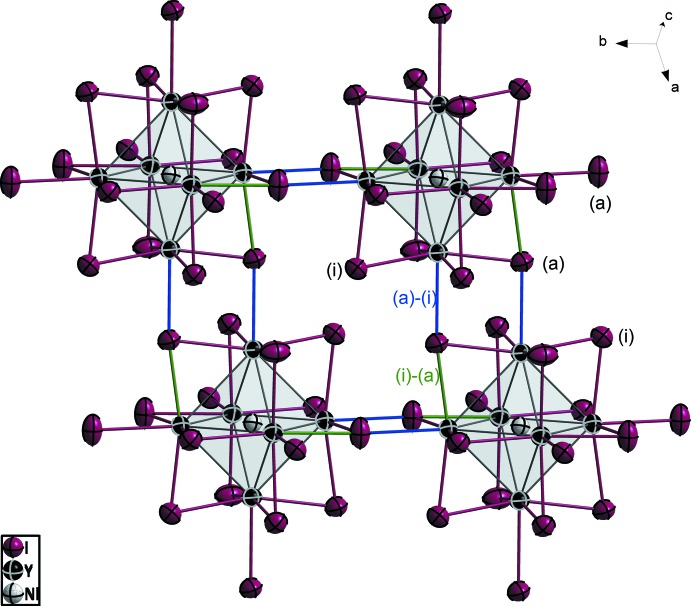
Representation of isolated [NiY6] octahedra. The edges are capped by the iodido ligands, whereas each yttrium atom bonds to one outer iodido ligand (90% probability thermal ellipsoids).

**Fig. 2.**
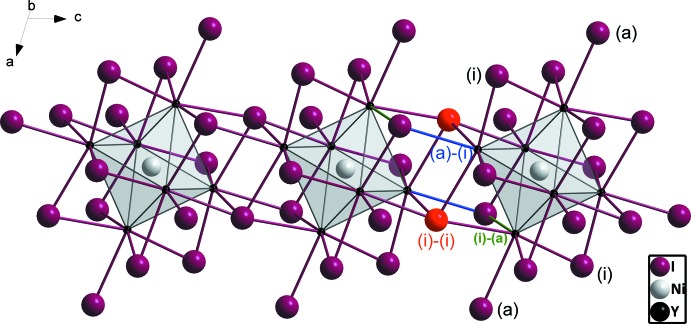
View on the (i)–(i)–, (i)–a)– and (a)–(i)–interconnections as seen in the triclinic [NiY6]I10.

## supplementary crystallographic information

### 1. Comment

[NiY_6_]I_10_ crystallizes with the triclinic [RuY_6_]I_10_ type of structure (Hughbanks *et al.*, 1989) and may be depicted as cubic closest packings of nickel and iodine atoms with yttrium atoms residing in 6/11 of all octahedral holes. Particularly, the yttrium atoms occupy those voids surrounding the nickel atoms to aggregate to octahedral [NiY_6_] clusters (Fig. 1). The twelve edges of each [NiY_6_] octahedron are capped by inner iodido ligands forming cuboctahedra around the endohedral nickel atoms. Additionally, the yttrium atoms bond to outer iodido ligands that reside in the inner coordination spheres of like clusters. More specifically, the iodido ligands interconnect the [NiY_6_] clusters *via* (i)–(i)–, (i)–(*a*)– and (*a*)–(i)–functionalities (Fig. 2), which can be emphasized by the formula [NiY_6_]I^i^_2/1_I^i-i^_4/2_I^i-a^_6/2_I^a-i^_6/2_ (Rustige *et al.*, 2012 and lit. cited therein). The averaged Y–Y and Y–I (see below) distances correlate well with data of recently reported yttrium cluster iodides (Rustige *et al.*, 2012).

### 2. Experimental

[NiY_6_]I_10_ was obtained from comproportionation reactions of yttrium triiodide, yttrium and nickel. Yttrium triiodide was synthesized from reactions of the pure elements (Corbett, 1983; Meyer, 1991), while the metals were obtained from commercial sources (Y, smart elements, 99.99%; Ni, Riedel–de Haen, 99.8%) and used without further purification. Due to the sensivity of the used chemicals and products to air and moisture, all sample preparations were performed under a nitrogen atmosphere in a glove box with strict exclusion of air and water (< 0.1 p.p.m.). The reaction mixtures were loaded as {NiY_3_}I_3_ in pre–cleaned, one–side He–arc welded tantalum tubes, which were closed inside a glove box, arc–welded at the other end and jacketed by evacuated, fused silica tubes. The mixtures were first heated to 1050 °C, kept at that temperature for one week, slowly–annealed to 700 °C and, then, rapidly cooled to room temperature. The product appeared as a black powder containing small crystals of polyhedral shape. Single crystals were selected from the bulk and fixed in capilleries, which were closed inside a glove box. The crystals were subsequently transferred to a Stoe *IPDS* 2 T diffractometer and complete sets of intensity data were collected at room temperature (293 (2) K).

### 3. Refinement

The intensity data sets were corrected for Lorentz and polarization effects. The structure was solved using direct methods (*SHELXS97*, Sheldrick, 2008) and refined on F^2^ (*SHELXL97*, Sheldrick, 2008). A numerical absorption correction and a crystal shape optimization were carried out with the programs *X-RED* (Stoe & Cie, 2001) and *X-SHAPE* (Stoe & Cie, 1999), respectively. The *PLATON* software package (Spek, 2009) was utilized to check the symmetry of the refined structure and no higher symmetry was identified. As the largest difference peaks (1.365 and -1.635 e.Å) are located 0.73 Å and 0.81 Å near to the I2 and I1 sites, respectively, and all thermal ellipsoids are reasonable in shape and size (Rustige *et al.*, 2012), the presence of further atom sites was excluded. To compare the lattice parameters of the title compound to those of more recently reported yttrium cluster iodides (Rustige *et al.*, 2012), a lattice setting unlike the standard setting was chosen for the triclinic [NiY_6_]I_10_.

### Crystal data

**Table:**

Y_6_NiI_10_	*Z* = 1
*M**_r_* = 1861.17	*F*(000) = 792
Triclinic, *P*1	*D*_x_ = 5.041 Mg m^−^^3^
Hall symbol: -P 1	Mo *K*α radiation, λ = 0.71073 Å
*a* = 9.4904 (7) Å	Cell parameters from 7675 reflections
*b* = 9.4990 (7) Å	θ = 2.3–29.7°
*c* = 7.5702 (5) Å	µ = 27.35 mm^−^^1^
α = 97.056 (6)°	*T* = 293 K
β = 105.096 (6)°	Polyhedral, black
γ = 107.540 (5)°	0.1 × 0.1 × 0.1 mm
*V* = 613.06 (8) Å^3^	

### Data collection

**Table:**

Stoe IPDS 2T diffractometer	3294 independent reflections
Radiation source: fine-focus sealed tube	2873 reflections with *I* > 2σ(*I*)
Graphite monochromator	*R*_int_ = 0.106
Detector resolution: not measured pixels mm^-1^	θ_max_ = 29.2°, θ_min_ = 2.3°
ω and φ scans	*h* = −12→12
Absorption correction: numerical [*X-SHAPE* (Stoe & Cie, 1999) and *X-RED* (Stoe & Cie, 2001)]	*k* = −12→12
*T*_min_ = 0.031, *T*_max_ = 0.084	*l* = −10→10
11699 measured reflections	

### Refinement

**Table:**

Refinement on *F*^2^	Primary atom site location: structure-invariant direct methods
Least-squares matrix: full	Secondary atom site location: difference Fourier map
*R*[*F*^2^ > 2σ(*F*^2^)] = 0.034	*w* = 1/[σ^2^(*F*_o_^2^) + (0.0426*P*)^2^] where *P* = (*F*_o_^2^ + 2*F*_c_^2^)/3
*wR*(*F*^2^) = 0.091	(Δ/σ)_max_ = 0.001
*S* = 1.05	Δρ_max_ = 1.37 e Å^−^^3^
3294 reflections	Δρ_min_ = −1.64 e Å^−^^3^
80 parameters	Extinction correction: *SHELXL*, Fc^*^=kFc[1+0.001xFc^2^λ^3^/sin(2θ)]^-1/4^
0 restraints	Extinction coefficient: 0.0065 (3)

### Special details

**Table:**

Geometry. All e.s.d.'s (except the e.s.d. in the dihedral angle between two l.s. planes) are estimated using the full covariance matrix. The cell e.s.d.'s are taken into account individually in the estimation of e.s.d.'s in distances, angles and torsion angles; correlations between e.s.d.'s in cell parameters are only used when they are defined by crystal symmetry. An approximate (isotropic) treatment of cell e.s.d.'s is used for estimating e.s.d.'s involving l.s. planes.
Refinement. Refinement of *F*^2^ against ALL reflections. The weighted *R*-factor *wR* and goodness of fit *S* are based on *F*^2^, conventional *R*-factors *R* are based on *F*, with *F* set to zero for negative *F*^2^. The threshold expression of *F*^2^ > σ(*F*^2^) is used only for calculating *R*-factors(gt) *etc*. and is not relevant to the choice of reflections for refinement. *R*-factors based on *F*^2^ are statistically about twice as large as those based on *F*, and *R*- factors based on ALL data will be even larger.

### Fractional atomic coordinates and isotropic or equivalent isotropic displacement parameters (Å^2^)

**Table:**

	*x*	*y*	*z*	*U*_iso_*/*U*_eq_	
I1	0.46133 (4)	0.27745 (5)	0.36198 (5)	0.03196 (12)	
I2	0.09092 (5)	−0.53117 (5)	−0.73275 (5)	0.03117 (12)	
I3	0.62560 (4)	0.18495 (5)	−0.08746 (6)	0.03022 (12)	
I4	0.18872 (4)	−0.09482 (4)	−0.45161 (4)	0.02376 (11)	
I5	0.26512 (4)	0.35524 (4)	−0.21790 (6)	0.02461 (11)	
Y1	0.03920 (6)	−0.24090 (6)	−0.88682 (7)	0.01994 (13)	
Y2	0.28566 (6)	0.08659 (6)	−0.02824 (7)	0.02070 (13)	
Y3	0.12577 (6)	0.16546 (6)	−0.65217 (7)	0.02003 (13)	
Ni1	0.0000	0.0000	0.0000	0.01763 (19)	

### Atomic displacement parameters (Å^2^)

**Table:**

	*U*^11^	*U*^22^	*U*^33^	*U*^12^	*U*^13^	*U*^23^
I1	0.0218 (2)	0.0379 (3)	0.02921 (19)	0.00438 (18)	0.00735 (15)	−0.00074 (17)
I2	0.0445 (3)	0.0225 (2)	0.02586 (18)	0.01531 (18)	0.00550 (16)	0.00573 (15)
I3	0.0238 (2)	0.0314 (2)	0.0443 (2)	0.01372 (17)	0.01644 (16)	0.01687 (19)
I4	0.02514 (19)	0.0264 (2)	0.02106 (17)	0.01056 (16)	0.00748 (13)	0.00530 (14)
I5	0.02516 (19)	0.0237 (2)	0.02650 (17)	0.00764 (14)	0.01043 (13)	0.00798 (13)
Y1	0.0214 (2)	0.0197 (3)	0.0214 (2)	0.00886 (18)	0.00798 (17)	0.00681 (18)
Y2	0.0188 (2)	0.0232 (3)	0.0223 (2)	0.00806 (18)	0.00821 (17)	0.00681 (19)
Y3	0.0212 (2)	0.0205 (3)	0.0198 (2)	0.00802 (18)	0.00750 (16)	0.00500 (17)
Ni1	0.0174 (4)	0.0184 (5)	0.0191 (4)	0.0072 (3)	0.0070 (3)	0.0060 (3)

### Geometric parameters (Å, º)

**Table:**

I1—Y3^i^	3.0062 (7)	Y1—Y2^vii^	3.7458 (8)
I1—Y2	3.0150 (7)	Y1—Y3	3.7913 (8)
I2—Y1^ii^	3.0839 (7)	Y2—Ni1	2.6595 (5)
I2—Y3^iii^	3.1124 (7)	Y2—I3^iv^	3.0999 (6)
I2—Y1	3.2421 (6)	Y2—Y3^i^	3.6579 (8)
I3—Y2^iv^	3.0999 (6)	Y2—Y1^vi^	3.7404 (8)
I3—Y1^v^	3.1255 (7)	Y2—Y1^i^	3.7458 (8)
I3—Y2	3.2437 (7)	Y2—Y3^vi^	3.8540 (8)
I4—Y1	3.1700 (6)	Y3—Ni1^vii^	2.6540 (5)
I4—Y3	3.1805 (6)	Y3—I1^vii^	3.0062 (7)
I4—Y3^vi^	3.1811 (7)	Y3—I2^ix^	3.1124 (7)
I4—Y2	3.1999 (6)	Y3—I4^vi^	3.1811 (7)
I5—Y1^vi^	3.1085 (7)	Y3—Y2^vii^	3.6579 (8)
I5—Y2	3.1091 (6)	Y3—Y1^viii^	3.6863 (7)
I5—Y3	3.2633 (7)	Y3—Y2^vi^	3.8540 (8)
Y1—Ni1^vii^	2.6339 (5)	Ni1—Y1^i^	2.6339 (5)
Y1—I2^ii^	3.0839 (7)	Ni1—Y1^vi^	2.6339 (5)
Y1—I5^vi^	3.1085 (7)	Ni1—Y3^vi^	2.6540 (5)
Y1—I3^v^	3.1255 (7)	Ni1—Y3^i^	2.6540 (5)
Y1—Y3^viii^	3.6863 (7)	Ni1—Y2^x^	2.6595 (5)
Y1—Y2^vi^	3.7404 (8)		
			
Y3^i^—I1—Y2	74.819 (17)	I1—Y2—Y1^vi^	95.963 (18)
Y1^ii^—I2—Y3^iii^	73.012 (17)	I3^iv^—Y2—Y1^vi^	142.723 (19)
Y1^ii^—I2—Y1	97.915 (17)	I5—Y2—Y1^vi^	53.010 (13)
Y3^iii^—I2—Y1	170.822 (19)	I4—Y2—Y1^vi^	95.321 (16)
Y2^iv^—I3—Y1^v^	73.981 (17)	I3—Y2—Y1^vi^	134.546 (18)
Y2^iv^—I3—Y2	97.455 (17)	Y3^i^—Y2—Y1^vi^	59.759 (14)
Y1^v^—I3—Y2	171.270 (19)	Ni1—Y2—Y1^i^	44.682 (11)
Y1—I4—Y3	73.313 (16)	I1—Y2—Y1^i^	96.665 (18)
Y1—I4—Y3^vi^	97.782 (17)	I3^iv^—Y2—Y1^i^	53.322 (14)
Y3—I4—Y3^vi^	92.193 (17)	I5—Y2—Y1^i^	141.452 (18)
Y1—I4—Y2	167.857 (18)	I4—Y2—Y1^i^	93.299 (17)
Y3—I4—Y2	97.453 (17)	I3—Y2—Y1^i^	135.838 (19)
Y3^vi^—I4—Y2	74.310 (16)	Y3^i^—Y2—Y1^i^	61.592 (15)
Y1^vi^—I5—Y2	73.966 (16)	Y1^vi^—Y2—Y1^i^	89.447 (15)
Y1^vi^—I5—Y3	97.326 (18)	Ni1—Y2—Y3^vi^	43.448 (11)
Y2—I5—Y3	97.588 (18)	I1—Y2—Y3^vi^	142.34 (2)
Ni1^vii^—Y1—I2^ii^	99.864 (17)	I3^iv^—Y2—Y3^vi^	93.585 (17)
Ni1^vii^—Y1—I5^vi^	97.898 (17)	I5—Y2—Y3^vi^	91.241 (16)
I2^ii^—Y1—I5^vi^	94.416 (18)	I4—Y2—Y3^vi^	52.622 (13)
Ni1^vii^—Y1—I3^v^	97.912 (18)	I3—Y2—Y3^vi^	133.462 (18)
I2^ii^—Y1—I3^v^	88.724 (19)	Y3^i^—Y2—Y3^vi^	89.881 (16)
I5^vi^—Y1—I3^v^	163.10 (2)	Y1^vi^—Y2—Y3^vi^	59.875 (14)
Ni1^vii^—Y1—I4	97.867 (18)	Y1^i^—Y2—Y3^vi^	58.011 (13)
I2^ii^—Y1—I4	162.27 (2)	Ni1^vii^—Y3—I1^vii^	99.248 (19)
I5^vi^—Y1—I4	83.191 (17)	Ni1^vii^—Y3—I2^ix^	98.706 (17)
I3^v^—Y1—I4	88.768 (17)	I1^vii^—Y3—I2^ix^	90.954 (19)
Ni1^vii^—Y1—I2	178.04 (2)	Ni1^vii^—Y3—I4	97.190 (17)
I2^ii^—Y1—I2	82.085 (17)	I1^vii^—Y3—I4	88.952 (18)
I5^vi^—Y1—I2	81.656 (16)	I2^ix^—Y3—I4	163.91 (2)
I3^v^—Y1—I2	82.341 (16)	Ni1^vii^—Y3—I4^vi^	96.629 (17)
I4—Y1—I2	80.185 (15)	I1^vii^—Y3—I4^vi^	164.07 (2)
Ni1^vii^—Y1—Y3^viii^	46.028 (12)	I2^ix^—Y3—I4^vi^	87.890 (18)
I2^ii^—Y1—Y3^viii^	53.849 (14)	I4—Y3—I4^vi^	87.807 (17)
I5^vi^—Y1—Y3^viii^	98.781 (17)	Ni1^vii^—Y3—I5	176.74 (2)
I3^v^—Y1—Y3^viii^	96.476 (18)	I1^vii^—Y3—I5	83.500 (17)
I4—Y1—Y3^viii^	143.879 (19)	I2^ix^—Y3—I5	82.942 (16)
I2—Y1—Y3^viii^	135.914 (19)	I4—Y3—I5	81.059 (15)
Ni1^vii^—Y1—Y2^vi^	45.319 (11)	I4^vi^—Y3—I5	80.592 (16)
I2^ii^—Y1—Y2^vi^	95.733 (17)	Ni1^vii^—Y3—Y2^vii^	46.558 (12)
I5^vi^—Y1—Y2^vi^	53.024 (13)	I1^vii^—Y3—Y2^vii^	52.700 (15)
I3^v^—Y1—Y2^vi^	143.204 (19)	I2^ix^—Y3—Y2^vii^	96.907 (18)
I4—Y1—Y2^vi^	96.770 (17)	I4—Y3—Y2^vii^	95.821 (17)
I2—Y1—Y2^vi^	134.455 (19)	I4^vi^—Y3—Y2^vii^	143.186 (18)
Y3^viii^—Y1—Y2^vi^	59.009 (14)	I5—Y3—Y2^vii^	136.194 (19)
Ni1^vii^—Y1—Y2^vii^	45.234 (12)	Ni1^vii^—Y3—Y1^viii^	45.581 (11)
I2^ii^—Y1—Y2^vii^	98.116 (18)	I1^vii^—Y3—Y1^viii^	97.251 (19)
I5^vi^—Y1—Y2^vii^	142.547 (19)	I2^ix^—Y3—Y1^viii^	53.138 (14)
I3^v^—Y1—Y2^vii^	52.697 (13)	I4—Y3—Y1^viii^	142.755 (19)
I4—Y1—Y2^vii^	94.288 (17)	I4^vi^—Y3—Y1^viii^	94.750 (17)
I2—Y1—Y2^vii^	134.926 (19)	I5—Y3—Y1^viii^	136.052 (19)
Y3^viii^—Y1—Y2^vii^	62.465 (15)	Y2^vii^—Y3—Y1^viii^	61.232 (14)
Y2^vi^—Y1—Y2^vii^	90.553 (16)	Ni1^vii^—Y3—Y1	43.984 (11)
Ni1^vii^—Y1—Y3	44.407 (11)	I1^vii^—Y3—Y1	95.871 (19)
I2^ii^—Y1—Y3	144.254 (18)	I2^ix^—Y3—Y1	142.674 (18)
I5^vi^—Y1—Y3	92.436 (17)	I4—Y3—Y1	53.216 (13)
I3^v^—Y1—Y3	94.680 (17)	I4^vi^—Y3—Y1	94.652 (16)
I4—Y1—Y3	53.471 (13)	I5—Y3—Y1	134.247 (18)
I2—Y1—Y3	133.650 (17)	Y2^vii^—Y3—Y1	60.346 (15)
Y3^viii^—Y1—Y3	90.435 (16)	Y1^viii^—Y3—Y1	89.565 (16)
Y2^vi^—Y1—Y3	61.551 (14)	Ni1^vii^—Y3—Y2^vi^	43.561 (12)
Y2^vii^—Y1—Y3	58.063 (14)	I1^vii^—Y3—Y2^vi^	142.796 (19)
Ni1—Y2—I1	98.905 (19)	I2^ix^—Y3—Y2^vi^	95.413 (17)
Ni1—Y2—I3^iv^	97.985 (17)	I4—Y3—Y2^vi^	94.365 (16)
I1—Y2—I3^iv^	90.836 (17)	I4^vi^—Y3—Y2^vi^	53.068 (13)
Ni1—Y2—I5	97.335 (17)	I5—Y3—Y2^vi^	133.637 (19)
I1—Y2—I5	95.806 (19)	Y2^vii^—Y3—Y2^vi^	90.119 (16)
I3^iv^—Y2—I5	162.14 (2)	Y1^viii^—Y3—Y2^vi^	59.523 (14)
Ni1—Y2—I4	96.070 (17)	Y1—Y3—Y2^vi^	58.574 (14)
I1—Y2—I4	165.00 (2)	Y1^i^—Ni1—Y1^vi^	180.0
I3^iv^—Y2—I4	86.140 (18)	Y1^i^—Ni1—Y3^vi^	88.391 (16)
I5—Y2—I4	83.167 (16)	Y1^vi^—Ni1—Y3^vi^	91.609 (16)
Ni1—Y2—I3	176.84 (2)	Y1^i^—Ni1—Y3^i^	91.609 (16)
I1—Y2—I3	84.194 (17)	Y1^vi^—Ni1—Y3^i^	88.391 (16)
I3^iv^—Y2—I3	82.545 (17)	Y3^vi^—Ni1—Y3^i^	180.00 (2)
I5—Y2—I3	81.665 (16)	Y1^i^—Ni1—Y2	90.083 (17)
I4—Y2—I3	80.844 (17)	Y1^vi^—Ni1—Y2	89.917 (17)
Ni1—Y2—Y3^i^	46.433 (12)	Y3^vi^—Ni1—Y2	92.991 (17)
I1—Y2—Y3^i^	52.481 (14)	Y3^i^—Ni1—Y2	87.009 (17)
I3^iv^—Y2—Y3^i^	97.823 (18)	Y1^i^—Ni1—Y2^x^	89.917 (17)
I5—Y2—Y3^i^	99.371 (18)	Y1^vi^—Ni1—Y2^x^	90.083 (17)
I4—Y2—Y3^i^	142.503 (18)	Y3^vi^—Ni1—Y2^x^	87.009 (17)
I3—Y2—Y3^i^	136.648 (19)	Y3^i^—Ni1—Y2^x^	92.991 (17)
Ni1—Y2—Y1^vi^	44.765 (11)	Y2—Ni1—Y2^x^	180.000 (7)

Symmetry codes: (i) *x*, *y*, *z*+1; (ii) −*x*, −*y*−1, −*z*−2; (iii) *x*, *y*−1, *z*; (iv) −*x*+1, −*y*, −*z*; (v) −*x*+1, −*y*, −*z*−1; (vi) −*x*, −*y*, −*z*−1; (vii) *x*, *y*, *z*−1; (viii) −*x*, −*y*, −*z*−2; (ix) *x*, *y*+1, *z*; (x) −*x*, −*y*, −*z*.

### Averaged Y—Ni, Y—Y and Y—I distances (Å) in triclinic [NiY_6_]I_10_

**Table:**

Interaction	Y—Ni	Y—Y	Y—I
Distance	2.649	3.746	3.143
